# Room Temperature NO_2_-Sensing Properties of N-Doped ZnO Nanoparticles Activated by UV-Vis Light

**DOI:** 10.3390/s25010114

**Published:** 2024-12-27

**Authors:** Angelo Ferlazzo, Giovanni Neri, Andrea Donato, Giovanni Gugliandolo, Mariangela Latino

**Affiliations:** 1 Department of Chemical Sciences, University of Catania, 95125 Catania, Italy; angelo.ferlazzo@unict.it; 2 Department of Engineering, University of Messina, 98166 Messina, Italy; giovanni.gugliandolo@unime.it; 3 Department of Engineering, Mediterranea University, 89122 Reggio Calabria, Italy; andrea.donato@unirc.it; 4 Consorzio Interuniversitario Nazionale per la Scienza e Tecnologia dei Materiali, 50121 Firenze, Italy; 5 CNR-IPCF, Institute for Chemical-Physical Processes Messina, 98158 Messina, Italy

**Keywords:** sol–gel, ZnO nanoparticles, N-doped ZnO, NO_2_ gas sensor

## Abstract

Zinc oxide nanoparticles (ZnO NPs) with varying levels of nitrogen (N) doping were synthesized using a straightforward sol–gel approach. The morphology and microstructure of the N-doped ZnO NPs were examined through techniques such as SEM, XRD, photoluminescence, and Raman spectroscopy. The characterization revealed visible changes in the morphology and microstructure resulting from the incorporation of nitrogen into the ZnO lattice. These N-doped ZnO NPs were used in the fabrication of conductometric gas sensors designed to operate at room temperature (RT) for detecting low concentrations of NO_2_ in the air, under LED UV-Vis irradiation (λ = 400 nm). The influence of nitrogen doping on sensor performance was systematically studied. The findings indicate that N-doping effectively enhances ZnO-based sensors’ ability to detect NO_2_ at RT, achieving a notable response (S = R/R_0_) of approximately 18 when exposed to 5 ppm of NO_2_. These improvements in gas-sensing capabilities are attributed to the reduction in particle size and the narrowing of the optical band gap.

## 1. Introduction

It is well established that sensors based on metal oxide (MOX) semiconductors can benefit from modifications to the sensing layer [1,2,3,4,5,6,7,8]. One commonly explored approach is doping MOX materials with electropositive elements (cations), as well as using metal and metal oxide modifiers. The mechanisms underlying these modifications are well understood [9,10,11,12]. However, less attention has been given to doping metal oxides with electronegative elements (anions), such as nitrogen (N) or fluorine (F), particularly for sensing applications. Nitrogen doping, in particular, is a promising strategy to alter the electrical properties of metal oxide semiconductors [13,14]. Nitrogen has a lower electronegativity compared to oxygen, a similar atomic size to oxygen in the lattice, and a low formation energy, making it a suitable candidate for substitutional doping within the crystal structure of MOX. The primary goal of nitrogen doping is to enhance the control over the physical, chemical, and electrical properties of the sensing layer, thereby improving the performance of MOX conductometric gas sensors.

Zinc oxide (ZnO), an n-type semiconductor with a wide direct band gap of 3.37 eV, possesses a high concentration of intrinsic defects such as oxygen vacancies and zinc interstitials. It has been extensively studied for MOX gas sensors due to its low cost, simple synthesis, non-toxic nature, high chemical and thermal stability, and tunable electrical properties [15,16,17,18,19]. In this study, we focus on nitrogen doping of ZnO with the aim of developing high-performance gas sensors. The ZnO sensing material was synthesized by a sol–gel process. It is well known that the main advantages of the sol–gel process are the high purity of the products, the narrow particle size distribution, and the achievement of uniform nanostructures at low temperatures [20].

Previous studies have shown that N-doping can influence the gas-sensing capabilities of ZnO-based sensors. For instance, Wen et al. reported that N-ZnO particles exhibited higher sensitivity to ethanol and acetone compared to two-dimensional porous ZnO flakes [21]. Similarly, Patil et al. explored the ethanol-sensing properties of N-doped ZnO films at 150 °C [22].

In the present work, we have developed a conductometric gas sensor platform based on ZnO and N-ZnO nanoparticles (NPs) designed to monitor low concentrations of NO_2_ in the air. NO_2_ is a highly toxic gas with a threshold limit value (TLV) of 5 ppm in ambient air, creating a demand for low-cost, highly sensitive, and selective NO_2_ sensors [15,16,17,18,19,20,21,22]. In a previous study [23], we investigated the impact of nitrogen doping on ZnO, showing promising results at elevated temperatures (from 200 °C to 350 °C). Moreover, it was observed that doping could reduce the operating temperature down to room temperature [24,25].

Based on these findings, we conducted a systematic study of NO_2_-sensing properties using N-doped ZnO NPs with varying nitrogen content to better understand the role of the dopant and optimize its loading for improved sensor performance. Our main objective is the synthesis of N-doped ZnO NPs with varying nitrogen content by a simple sol–gel technique and the monitoring of nitrogen dioxide at room temperature. Both these objectives are the main requirements when designing a sensor for practical application in the environmental field, due to the constraints required for mass production and minimal power consumption of the sensors.

## 2. Materials and Methods

This section provides a detailed description of the procedures followed for the synthesis of the sensing material, the morphological analysis of the sensor substrate, and the performance testing of the fabricated sensors. First, the sensing material synthesis process is presented with a description of the sol–gel method used to prepare ZnO and N-doped ZnO nanoparticles. This is followed by an examination of the sensor substrate, including measurements of the thickness, width, and spacing of the platinum electrodes and the integrated heater. Finally, the methodology for the gas-sensing tests is outlined, detailing the experimental setup, test conditions, and approach used to evaluate the sensor response to NO_2_.

### 2.1. Sensing Material Synthesis

**Figure 1:**
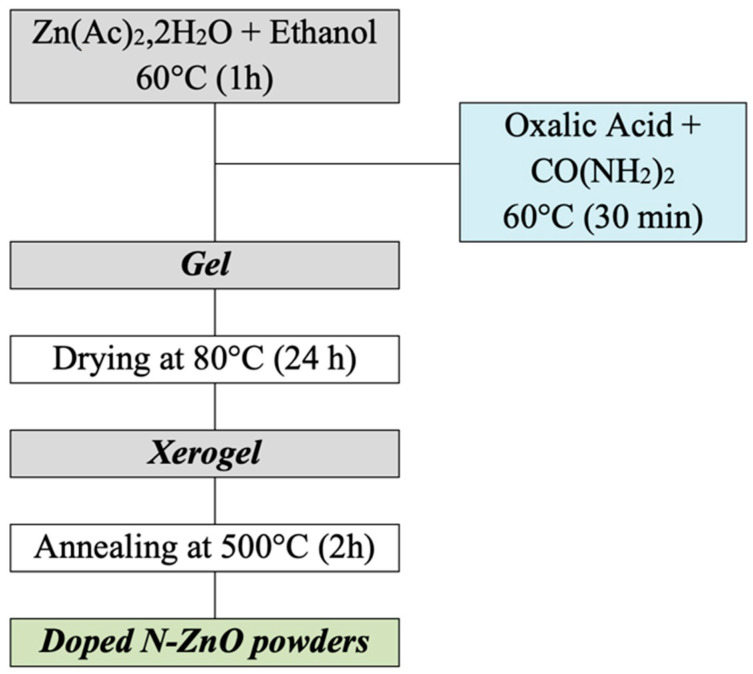
Flowchart of the synthesis of the doped ZnO NPs.

ZnO was synthesized through a sol–gel method, using zinc acetate dihydrate (Zn[CH_3_COO]_2_·2H_2_O; Lobachemie) as the metal precursor, ethanol (C_2_H_6_O; AnalaR NORMAPUR) as the solvent, and oxalic acid dihydrate (C_2_H_2_O_4_·2H_2_O; SIGMA-ALDRICH) as the complexing agent. To create nitrogen-doped ZnO (N-ZnO) samples, urea (CO(NH_2_)_2_) was employed as the nitrogen precursor, with molar ratios of 0%, 3%, 5%, and 7% (Figure 1). The synthesized ZnO and N-ZnO samples were labeled as ZnO and xN-ZnO, respectively, where “x” denotes the nominal nitrogen content.

Scanning electron microscopy (SEM) imaging was performed using a Zeiss Crossbeam 540 instrument (ZEISS: Oberkochen, Germany), operating at acceleration voltages between 3 kV and 20 kV. X-ray diffraction (XRD) analysis was conducted on powdered samples with a Bruker D8 Advance Diffractometer, utilizing CuKα1 radiation (λ = 1.5405 Å). Data were collected over a 2Θ range of 20–80°. Diffraction peak identification was conducted using the JCPDS reference compound database. Photoluminescence (PL) measurements were carried out at room temperature using a Horiba NanoLog modular spectrofluorometer (HORIBA: Bangkok, Thailand), equipped with a xenon lamp as the excitation source, with an excitation wavelength of 340 nm. Raman spectroscopy was conducted at room temperature in ambient air using a Horiba XploRA spectrometer (HORIBA: Bangkok, Thailand). Raman spectra were recorded over the range of 100–600 cm^−1^ and subsequently normalized.

### 2.2. Sensor Substrate Morphological Study

To better characterize the morphology of the sensor substrate, a detailed profile analysis of the interdigitated platinum electrodes and the heater embedded in the alumina substrate was performed using a DektakXT-Bruker profilometer (Bruker: Billerica, MA, USA). The DektakXT system conducts measurements electromechanically by moving a diamond-tipped stylus over the sample surface, with user-programmable scan length, speed, and stylus force. This system can handle samples up to 50 mm thick and perform scans up to 55 mm long. The stylus tip has a diameter of 2 microns. The aim of the measurements was to evaluate the thickness, width, and spacing of the platinum tracks, providing a more comprehensive understanding of the sensor’s geometrical characteristics and the uniformity of the platinum layers.

**Figure 2:**
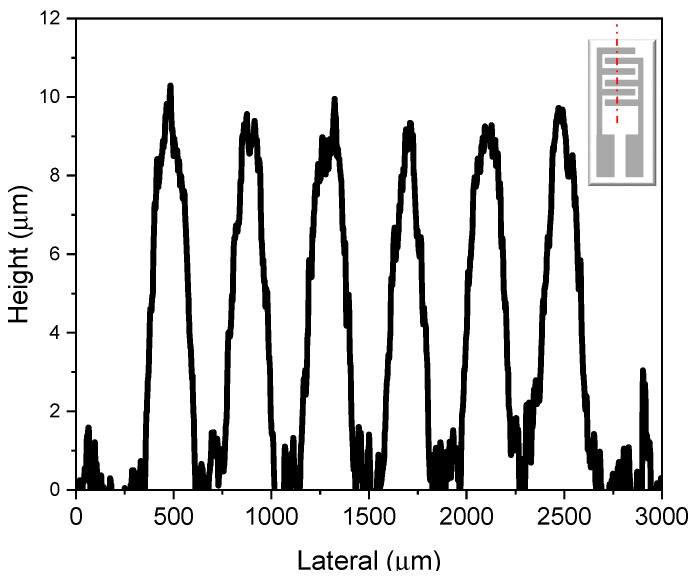
Results from the profilometry measurements along the path indicated (red dots) in the inset at the upper right of the plot.

For this study, a blank substrate was used, i.e., the alumina substrate before the deposition of the sensing material, with both the heater and interdigitated electrodes exposed. Using the DektakXT profilometer, five distinct paths were scanned across the interdigitated electrodes on the front side of the substrate, and an additional five paths were analyzed across the heater located on the backside. Figure 2 shows a plot of the results obtained from the DektakXT profilometer across one of the selected paths on the interdigitated structure (red dots), shown in the inset in Figure 2.

From this plot, the average thickness of the platinum layer, as well as the geometrical dimensions along the profile path, can be extracted. The mean and standard deviation for each geometrical feature were calculated to assess the reliability and uniformity of the substrate. The results are summarized in Table 1.

The morphological study shows that the fingers of the interdigitated electrodes have a thickness of 7.2 µm ± 0.8 µm, a width of 199 µm ± 15 µm, and a spacing of 198 µm ± 11 µm. The first number represents the average value, and the second indicates the standard deviation. Considering the nominal thickness of 10 µm and a nominal width and spacing of 200 µm for the alumina substrate, the studied sample exhibited geometrical dimensions close to the nominal ones, with good uniformity, as indicated by the low standard deviations obtained from multiple measurement paths.

Similarly, the heater on the backside of the substrate was evaluated, yielding a mean platinum thickness of 7.6 µm ± 0.6 µm, a width of 212 µm ± 29 µm, and a spacing of 206 µm ± 12 µm. The results for the heater were also close to the nominal values, with overall uniformity remaining within acceptable levels.

### 2.3. Sensor Test

**Figure 3:**
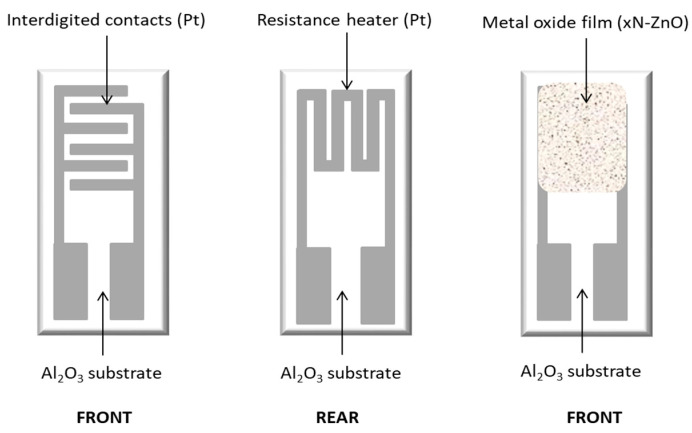
Sensor device before and after the deposition of the sensing material on the top side of the interdigitated electrodes.

For gas-sensing applications, conductometric sensors were fabricated by screen-printing thick ZnO films onto alumina substrates (6 mm × 3 mm) fitted with platinum interdigitated electrodes and a platinum heater on the reverse side (Figure 3) [26].

The sensing tests were conducted using a custom-built apparatus, which included a stainless-steel test chamber housing the sensor and was connected to the necessary gas and power supplies.

During the sensing tests, the sensors were placed in a test chamber and, after a stabilization period at the target working temperature in a synthetic dry air flow, they were exposed to pulses of a gas mixture. The concentration of NO_2_ varied from 140 ppb to 5 ppm. The sensing devices were interfaced with a PC, enabling the setting and control of the working temperature (ranging from 30 to 100 °C) and the measurement of the sensor’s resistance values using a flow of NO_2_/air mixture (100cc/min). The sensor response (S) was defined as “S = R/R_0_” for NO_2_, where R represents the sensor electrical resistance at a given NO_2_ concentration, and R_0_ is the baseline resistance in dry synthetic air. Additionally, dynamic sensor characteristics such as response time (τ_res_)—the time taken for the sensor resistance to reach 90% of its equilibrium value after NO_2_ exposure—and recovery time (τ_rec_)—the time required for the sensor resistance to return to 90% of its baseline value in air—were evaluated.

## 3. Results and Discussion

### 3.1. Morphological and Microstructural Characterization

**Figure 4:**
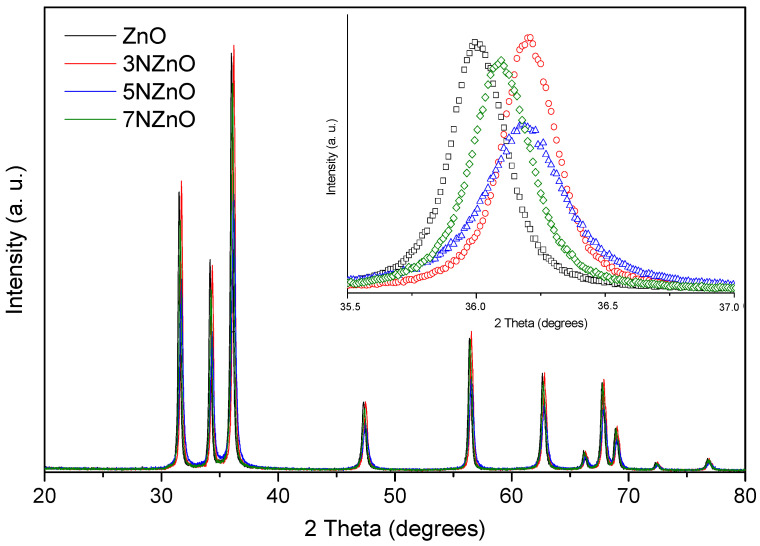
X-ray diffraction spectra of ZnO NP samples.

The structural characteristics of the synthesized samples were further analyzed using XRD (Figure 4). All samples exhibited the hexagonal wurtzite structure characteristic of zinc oxide (JCPDS No. 36-1451), as confirmed by the diffraction peaks corresponding to the (100), (002), (101), (102), (110), (103), and (112) planes of ZnO. These findings suggest that the introduction of nitrogen does not alter the crystal structure of ZnO. A magnified view of the diffraction patterns reveals a noticeable red shift in the (101) peak position in the doped samples, indicating successful nitrogen incorporation into the ZnO lattice [27]. We can note that both the shift and intensity reduction in the diffraction peaks do not follow a clear trend with the N-doping. This suggests that the introduction of N into the ZnO lattice is not straightforward, depending in a complex manner on the N:ZnO ratio and leading to samples with different structural parameters and different distortion degrees.

The average crystallite size, d, was determined using Scherrer’s formula:

D = (0.9λ)/(BcosΘ_B_), (1)

where λ is the X-ray wavelength of the radiation used (CuKα1 = 1.5405 Å), θ_B_ is the maximum of the Bragg diffraction peak (in radians), and B is the full width at half maximum (FWHM) of the XRD peak. The calculated crystallite size ranges from 26 nm to 38 nm, which is smaller than the size observed via SEM. This discrepancy suggests that the grains observed in the SEM images are polycrystalline in nature.

**Figure 5:**
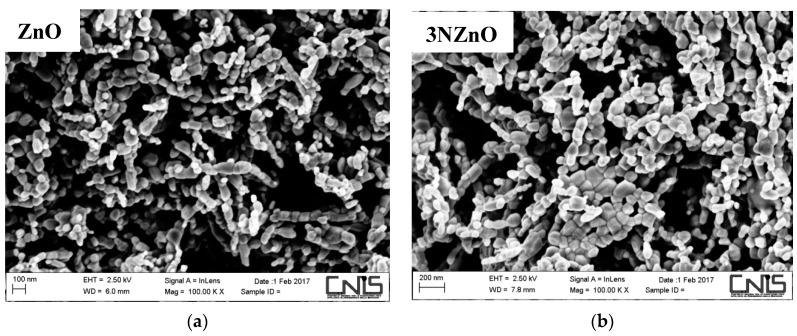

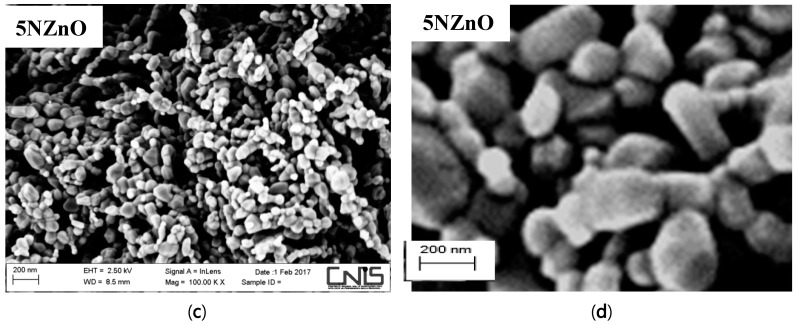
SEM images of N-doped ZnO samples: (**a**) ZnO; (**b**) 3NZnO; (**c**) 5NZnO; (**d**) 5NZnO.

SEM images in Figure 5 display the morphology of the nitrogen-doped sample (xN-ZnO). The particles exhibit an irregular shape, typical of nanoparticles. A closer look reveals that the overall morphology remains consistent across the samples, indicating that nitrogen doping does not significantly affect the morphological features of the original ZnO NPs. Compared to XRD, the SEM technique (see Figure 5d) shows a larger particle size instead, indicating that they are constituted by these smaller primary crystallites.

**Figure 6:**
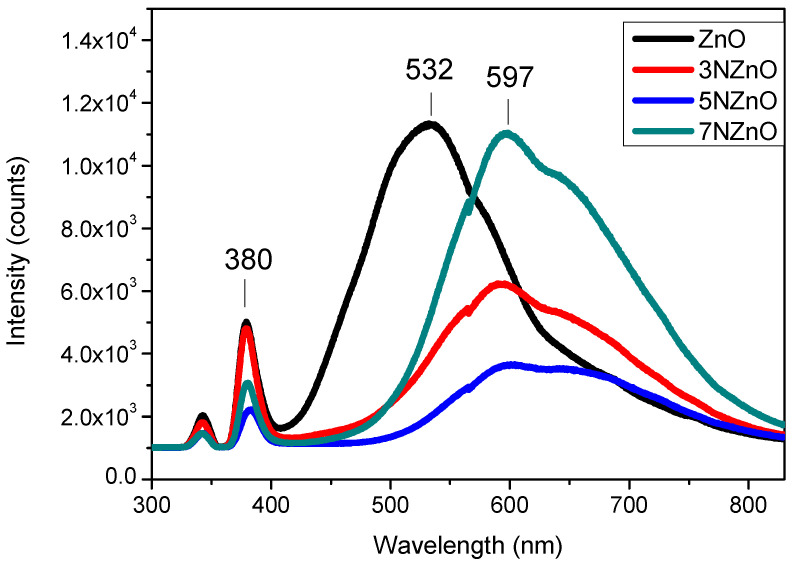
PL spectra of ZnO NP samples.

Photoluminescence (PL) spectroscopy, which is useful for assessing defects in metal oxide semiconductors, was employed to evaluate the synthesized samples. Figure 6 shows the PL emission spectra, acquired at room temperature, of the samples investigated.

The pure ZnO nanoparticles exhibit two main emission peaks: a near-band-edge (NBE) emission in the UV region (around 380 nm) attributed to the recombination of electrons and holes at the band edges and a broader, stronger green emission peak around 532 nm [28]. The visible emission is commonly associated with oxygen vacancies on the ZnO surface. On N-doped ZnO samples, the large green band shift is around 532 nm vs. higher wavelengths (597 nm). This shift of visible emission band on N-doping may be due to changes in the local environments of the defect centers in the samples [29]. Furthermore, we also noted a change in the intensity of the blue and green peaks, confirming the modifications of the structural parameters with the N-doping.

**Figure 7:**
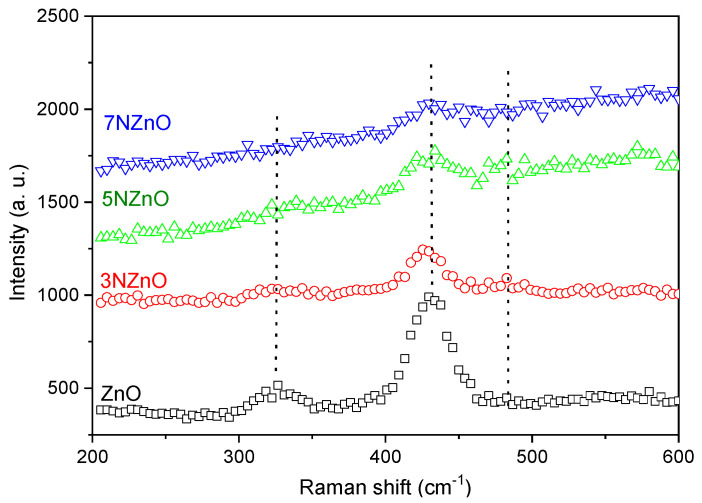
Raman spectra of ZnO NP samples.

Raman spectra, shown in Figure 7, provide further insight. In the undoped ZnO sample, characteristic peaks were observed at 333 cm^−1^ and 438 cm^−1^, corresponding to the E2 (high) vibrational modes [30]. The prominent peak at 438 cm^−1^ is a hallmark of the Raman-active E2 (high) mode of wurtzite ZnO [31], while the peak at 333 cm^−1^ corresponds to a second-order vibrational mode, specifically E2 (high)–E2 (low).

The particle size (d_XRD_), as measured using XRD, and the optical band gap energy (Eg) determined through UV-Vis measurements are detailed in Table 2. The band gap energies reported in Table 2 were obtained using the Tauc formula:

(αhν)^2^ = A(hν − Eg) (2)

where A is a constant, and Eg is the band gap energy. It is well known that variation in the band gap with particle sizes is due to different chemical and structural parameters, e.g., phase, strain, and particle sizes [32]. Data for the relationship between the band gap values and the crystallites indicate that the band gap increases with the crystallite’s sizes. Generally, the band gap energy increases with decreasing particle size because of the quantum size confinement. In our case, this effect could be neglected because of the relatively large size of the crystallite samples, whereas the other factors above mentioned and linked to the N-doping could give a main contribution. With the N-doping increase, the band gap energy first decreases and then increases. The significance of these characteristics in relation to the gas-sensing performance of the samples will be outlined below.

### 3.2. Sensing Tests

MOX gas sensors typically operate at elevated temperatures, ranging from 200 °C to 400 °C, leading to high power consumption. However, by using light irradiation, measurements can be conducted at lower temperatures, significantly reducing power requirements and enabling battery-operated devices suitable for field applications [33]. ZnO, one of the most extensively studied sensing materials, has garnered considerable attention because UV light exposure is known to reduce ZnO baseline resistance, likely due to the generation of free charge carriers.

**Figure 8:**
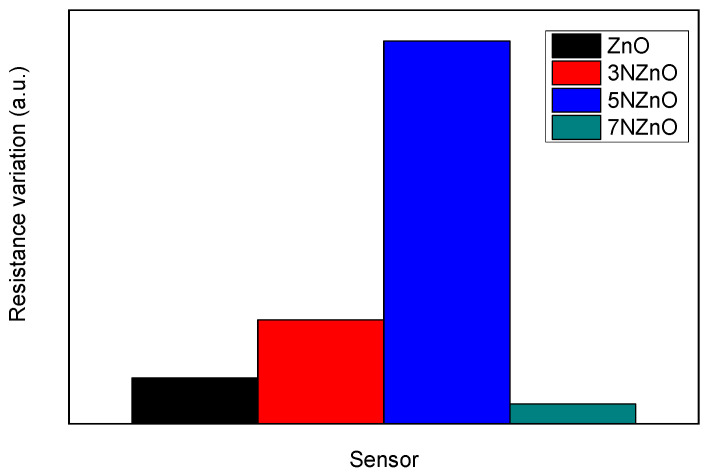
Baseline resistance variation in N-doped ZnO sensor under UV irradiation.

In this study, we explored the effect of UV-Vis light (λ = 400 nm) on the baseline resistance (Figure 8).

As the results show, ZnO is photoactivated under UV light, which reduces the baseline resistance. However, this reduction is more pronounced in N-doped ZnO samples. Among these, the 5N-ZnO sensor exhibited the best performance, likely due to its lower band gap compared to the other tested materials (see Table 1). In semiconductor materials with a smaller band gap, it is easier for valence band electrons to be excited to the conduction band under light exposure, increasing conductivity. The decrease in optical band gap energy resulting from nitrogen doping may have led to the formation of defects and an enhanced flow of electrons from the valence to the conduction band, thus improving the electronic conductivity of the doped sensor material.

**Figure 9:**
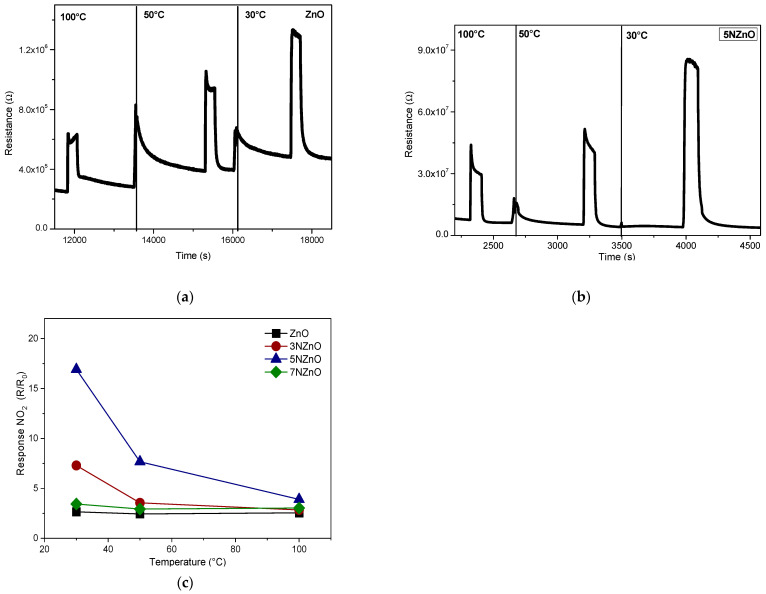
Tests of the ZnO (**a**) and 5N-ZnO (**b**) sensors vs. 5 ppm of NO_2_ under UV irradiation at different low temperatures from 30 to 100 °C. Response vs. temperature of the developed samples when exposed to 5 ppm of NO_2_ (**c**).

In previous work, we evaluated the effect of UV-Vis light (λ = 400 nm) on the detection of low NO_2_ concentrations in the air using pure N- and F-doped ZnO sensors [23]. Here, we focus on the performance of the N-doped ZnO sensor series, comparing their sensing behavior under UV illumination. We analyzed the variation in resistance in response to 5 ppm pulses of NO_2_ at different temperatures under light activation. The results for ZnO and 5N-ZnO sensors are presented in Figure 9a,b.

Generally, the response of the MOS sensor as a function of the temperature follows a volcano curve, i.e., initially, the response increases with the temperature, reaches a plateau, and then decreases. This behavior originated from the complex adsorption/reaction/desorption processes which occur on the surface due to the interaction of the target gas with the sensing material. In our case, these processes are likely strongly activated by UV light, and then the plateau is reached at near room temperature, so we observed only the descending part.

Based on the observation of the sensing tests, the sensing mechanism upon exposure to NO_2_ can be associated with the following reaction:

NO_2(g)_ + e^−^ → NO_2_^−^_(ads)_

This relies on the NO_2_ molecule’s ability to withdraw electrons from the semiconductor surface, causing the depletion layer’s thickness to further rise. Conduction electrons, e^−^, are then consumed, and this leads to an increase in surface resistance, in agreement with the experimental results reported.

The data revealed several key findings: (i) N-doped ZnO sensors outperform undoped ZnO sensors, (ii) lower operating temperatures result in improved sensor responses to NO_2_, and (iii) the 5N-ZnO sensor exhibited the best overall performance, as shown in Figure 9c.

**Figure 10:**
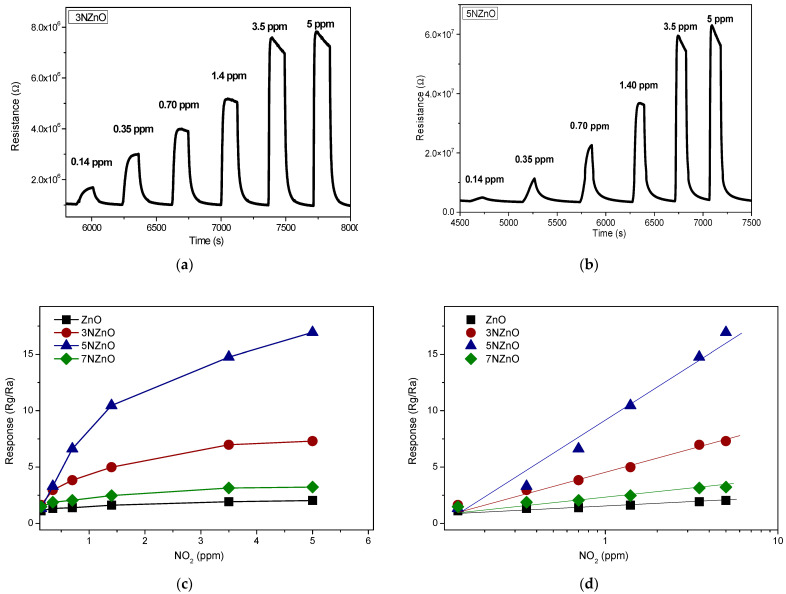
Tests of the ZnO (**a**) and N-ZnO (**b**) sensors vs. different concentration values of ppm of NO_2_ under UV irradiation at temperature value of 30 °C. Response vs. NO_2_ concentration values of the developed sensors under UV irradiation at temperature value of 30 °C (**c**,**d**).

Based on these results, we conducted further tests under UV illumination at an operating temperature of 30 °C, examining the response of the sensors to varying concentrations of NO_2_. The dynamic response of several N-doped ZnO sensors to NO_2_ under UV light is shown in Figure 10.

For all samples, a reversible change in resistance was observed upon exposure to NO_2_, followed by recovery when purged with clean air (Figure 10a,b).

The response, taken as the ratio between the resistance in the gas at a certain concentration and the resistance baseline in the air, follows a linear trend at low concentrations, and then a saturation is reached at higher concentrations (Figure 10c), as generally observed for MOS semiconductor sensors based on metal oxides. If the response–concentration curve follows the ideal Langmuir behavior, a linear trend can be observed plotting the data as a function of the logarithmic concentration (Figure 10d).

**Figure 11:**
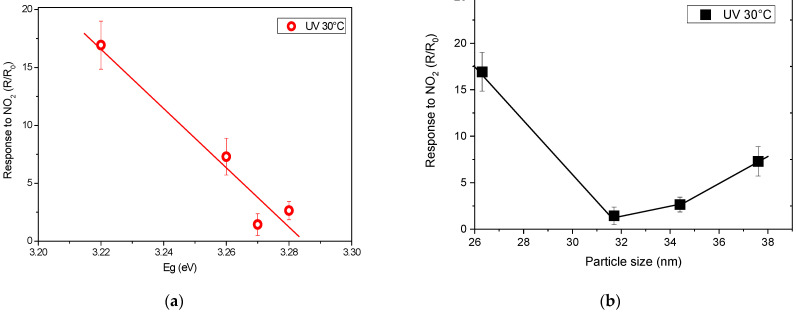
Plot reporting the trend of sensor responses to NO_2_ (5 ppm) at temperatures of 30 °C under UV light irradiation vs. (**a**) the optical band gap and (**b**) grain size.

To interpret these findings, we should consider that, under dark conditions, the sensing properties primarily depend on the morphology and microstructure of the sensing layer. Nanostructured materials with small particle sizes and high surface-to-volume ratios generally exhibit greater sensitivity. These factors also influence the sensor performance under UV illumination, along with the optical band gap. From the analysis of particle size and band gap values, it becomes clear that the 5N-ZnO sensor shows the highest sensitivity to NO_2_. We attempted to establish a correlation between these properties and the sensor response (Figure 11).

The results clearly indicate that the sample with the smallest particle size and lowest optical band gap (5N-ZnO) is the most sensitive to NO_2_ at room temperature under the conditions tested. Additionally, Figure 11 suggests that the optical band gap is a crucial factor influencing sensor response. The sensor response trends with respect to the optical band gap (Eg) are well defined, whereas the particle size trends are less consistent, with some unexpected increases in response as particle size increases.

## 4. Conclusions

Nitrogen-doped ZnO powders were successfully synthesized using a straightforward sol–gel method. The N-doped ZnO sensors demonstrated superior gas-sensing performance for NO_2_ detection compared to undoped ZnO. UV-Vis irradiation enabled operation at room temperature with rapid response dynamics. The enhancements in gas-sensing performance can be attributed to the reduction in particle size and the narrowing of the optical band gap, achieved through an optimal nitrogen doping of 5% in the ZnO nanostructure.

## Figures and Tables

**Table 1:** Average values and standard deviations for the thickness, width, and spacing of the interdigitated electrodes and heater on the sensor substrate.

	Interdigitated Electrodes		Heater	
	Mean (µm)	Standard Deviation (µm)	Mean (µm)	Standard Deviation (µm)
Thickness	7.2	0.8	7.6	0.6
Width	199	15	212	29
Spacing	198	11	206	12

**Table 2:** XRD particle sizes (d_XRD_) and band gap energy (E_g_) of N-doped ZnO samples.

Samples	d_XRD_ (nm)	Eg (eV)
ZnO	34.4	3.28
3NZnO	37.6	3.26
5NZnO	26.3	3.22
7NZnO	31.7	3.27

## Data Availability

The data can be considered available on specific request to all authors by any interested reader.
